# Do I Know You? How Individual Recognition Affects Group Formation and Structure

**DOI:** 10.1371/journal.pone.0170737

**Published:** 2017-01-26

**Authors:** Vitor Passos Rios, Roberto André Kraenkel

**Affiliations:** 1 Programa de Pós-Graduação em Ecologia, Universidade de São Paulo, São Paulo, São Paulo, Brazil; 2 Instituto de Física Teórica, Universidade Estadual Paulista, São Paulo, São Paulo, Brazil; Consejo Nacional de Investigaciones Cientificas y Tecnicas, ARGENTINA

## Abstract

Groups in nature can be formed by interactions between individuals, or by external pressures like predation. It is reasonable to assume that groups formed by internal and external conditions have different dynamics and structures. We propose a computational model to investigate the effects of individual recognition on the formation and structure of animal groups. Our model is composed of agents that can recognize each other and remember previous interactions, without any external pressures, in order to isolate the effects of individual recognition. We show that individual recognition affects the number and size of groups, and the modularity of the social networks. This model can be used as a null model to investigate the effects of external factors on group formation and persistence.

## Introduction

### Group living

Animal aggregations have long interested researchers. The idea of several animals living in close proximity seem, at first glance, counter intuitive: proximity can bring with it a host of disadvantages [1]: increased chance of pathogens transmission, increased visibility to predators, increased demand for scarce resources, increased chance of intraspecific competition, and even reduced direct fitness [2], for instance. However, under some circumstances, aggregations can be beneficial for an individual, if not for the whole group. For instance, there can be safety in numbers: even though a large group is more visible than a single individual is, the probability of any single individual being predated is decreased when there are many others from which to choose [3]. Group living also allows for individuals to share resources when these are scarce or unevenly distributed, and for better vigilance against predators or competitors [4]. Group living can also lead to more cost-efficient burrowing [5]. Large aggregations of animals can happen without visible direct interactions other than mere proximity [3], and these aggregations can display quite intricate collective behavior, even in the absence of perceived communication [6]. Groups can also be highly compact and highly interactive, as in colonies of social rodents, where the individuals share resources and interact frequently [7]. These groups can be quite large, or comprise only a breeding pair and its offspring. There are also fission-fusion societies, in which social bonds can last for a long time, that is, repeated interactions are frequent between individuals, but in which spatial relationships are often not constant, with individuals frequently separating (fission) only to regroup later (fusion) (e.g., bats [8]).

Current models of sociality rarely take movement or space into account (see for instance [9, 10]), but animal interactions happen in space, and, barred a few sessile groups, animals are motile. These characteristics shape the interactions and their consequences: animals can leave groups to forage and return, or can be forced to leave permanently due to agonistic interactions. Groups can also defend home ranges and share resources, and it has been shown that space is an important factor in the evolution of cooperation [11].

These different kinds of groups give rise to several questions: if groups can be induced externally (*e.g.*, by density constraints or predator pressure) or internally (*e.g.*, by generation overlap), how, if at all, do repeated individual interactions alter the group’s structure? Do preferential interactions lead to different group dynamics? Do groups based on repeated interactions last longer than groups formed by density pressures?

For repeated interactions between a pair of individuals to have an effect different from a series of random encounters, it is necessary that the pair in question react to each other differently than they would react to a stranger. In other words, memory, in the form of recognition, must exist. Recognition can be as specific as *individual recognition (IR)*, in which an individual associates a particular conspecific with some specific information [12], or as general as *class-level recognition*, where the information is associated with a certain trait that can be shared by many conspecifcs, *e.g.*, a colony odor, as in honeybees [13].

We seek to investigate how do memory and individual recognition affect group structure and stability, by using a computer simulation approach. Using a spatially explicit model, we can investigate the effects of individual recognition in repeated interactions with movement in space, which will help us answer our questions. We expect that simulations with individual recognition will give rise to groups with different characteristics and durations than simulations without it.

## Methods

In this paper, we define *group* as “a spatial aggregation of conspecifics”, regardless of presence or absence interactions between the individuals it comprises. We choose to use a purely spatial definition as we intend to study the effects interaction have on grouping, and including interaction directly on the definition would be troublesome for this purpose. We purposefully avoid using terms like *society, colony, band, flock* and others as they are loaded with meaning, and can imply a defined group structure. *Group structure* here refers to “the pattern of social behaviors between individuals in the group” and *group stability* is “group persistence trough time”.

### How to investigate memory?

We chose to approach our question using a modelling technique called Agent-Based Modelling. Agent-Based models (ABMs, [14]), also called individual-based models, are uniquely suited to problems where individual variation is key. In this type of model, entities such as individuals are represented as agents. This allows each individual in the model to have unique histories and behaviors, which means that each agent can have, for instance, its own genetic code (*e.g.*, [15]), or its own perception of the world, which allow for emergence of macroscopic properties from microscopic mechanisms [16]. ABMs allow for agents to represent individuals of different species, with differing ecological traits and resource requirements. ABMs are also well-suited to investigate mechanistic models, by representing entities with defined interactions and internal processes. Agent-based models have successfully been used to model certain aspects of animal societies, like hierarchies. These kinds of characteristics would be extremely difficult to model with traditional mathematical tools, which are more suited to homogeneous entities with regular behavior.

These characteristics make ABMs ideal for modelling memory, as memory is nothing more than an individual’s particular history. ABMs also allow for perfect control of the virtual experiments, eliminating possible sources of confusion, such as resource distribution and competition from other species. By running simulations identical except for presence or absence of memory, we are able to isolate any possible effects memory has. ABMs can easily model the effects of space and movement on social interaction [17]. Here we use an ABM to model individual interactions and to monitor group formation and changes on group structure in a homogeneous space. To isolate the effects of memory, we compare a simulation memory and one without, without external confounding factors.

### The model

**Fig 1:**
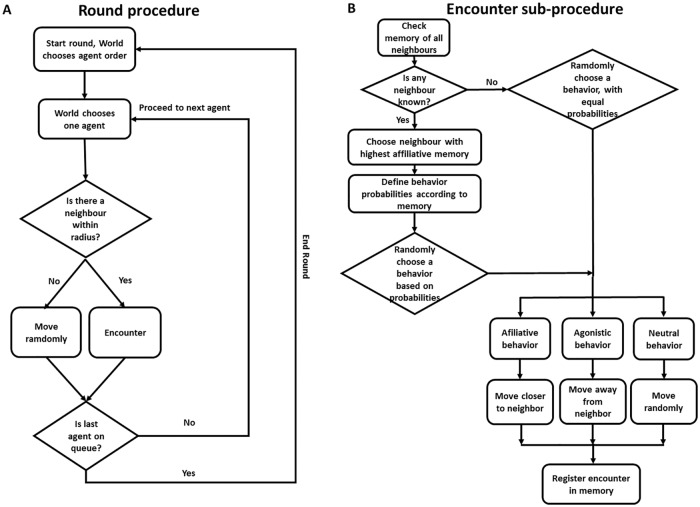
Simplified fluxogram of the model. Panel A shows the order of events in each round. Panel B shows the encounter procedure.

We refer the reader to S1 Text for the model’s full description and ODD protocol [18]. Here we give a brief explanation of how our model works. The full code of the model is available at https://github.com/vrios/SocS. The individuals, called *agents*, exist in a world without physical obstacles or constraints other than world size. The general model is summarized in Fig 1. Each agent moves freely in a random walk, until it encounters another agent. The result of this encounter is a behavior that depends on the agents’ memory: they have a higher probability of repeating previous behaviors than of engaging in different ones. In other words, if they have had more affiliative encounters than agonistic ones, they probably will have another affiliative encounter, and vice-versa. If the two agents have not encountered each other previously, one behavior (agonistic, affiliative or neutral) is chosen randomly, with equal probabilities for each kind. The behaviors are represented by movement in the model: in an affiliative encounter, the acting agent moves closer to the other one, while in an agonistic encounter, it moves away. In a neutral encounter, the agent moves randomly.

These three types of behavior were chosen to represent the three possibilities animal have when meeting a conspecific: to act affiliatively (cooperating or allogrooming, for instance), to act agonistically (territorial displays or fights), or to ignore each other. We vary the magnitude of the effect memory has by changing the durations of memory from remembering zero encounters to remembering 30 encounters with each other agent, and by varying the intensity of the effect individual recognition has on the behaviors. The probability of engaging in the same type of behavior increases by a given amount with each interaction, and this amount ranges from 0.5% to 50% per interaction.

## Analysis

We analyze the resulting groups from two different perspectives, spatial and social. Since our definition of group is spatial, we use a spatial clustering algorithm, DBSCAN [19], to investigate whether simulations with IR result in different spatial patterns. DBSCAN gives us the number, size and composition of spatial groups. This allows us to compare whether groups survive in time using the MONIC algorithm [20], which compares group composition in successive moments. Thus, our spatial metrics are number of groups, average group size, and average group lifespan. We predict that these three metrics will all be larger in simulations with IR.

These metrics tell us about the aggregation, but do not inform us about how the groups formed by, for example, spatial constraints, differ from those formed by social interactions. It is to be expected that higher densities lead to larger group sizes, but this does not mean that the individuals in those groups interact with each other. To examine the social consequences of IR we use social network analysis [21]. Social networks can be used to describe interactions between individuals in the form of graphs, with individuals as nodes and interactions as edges, with stronger interactions having higher edge weights. Differences in connectivity and connection strength can give rise to a modular network. Modular networks are networks which are divided in subsets, called modules, in which nodes are more strongly connected to each other than to nodes outside of these subsets [22].

In our model an edge is formed whenever two agents interact, and the weight depends on the type of interaction: -1 for agonistic, 0 for neutral and +1 for affiliative. The sum of all interactions, positive and negative, between two agents determines the final weight of the edge, and thus describes the prevailing type of interaction between those two agents. Individuals in close proximity will have a higher probability of interacting with each other than with distant individuals. If the type of interaction is based on previous interactions, we should see a higher modularity than if the interactions happen randomly. We calculate modularity using the Louvain algorithm [23], taking into account only the positive weights of the edges at the end of the simulations, that is, the modules are based only on primarily affiliative interactions.

Since our models have a strong probabilistic component, metrics are presented as the average of 100 replicates with the same starting conditions, run for 1000 rounds. Simulations and DBSCAN were written in C++11 using the QT 5.0 framework (http://www.qt.io/). Analyses were made in R version 3.2.1 [24] using the Igraph package [25], and MONIC version 1.0 [20]. We used parameter values for DBSCAN of *Epsilon* = 3 and *MinPts = 4*, the values recommended in literature for clustering in 2d space [19] Simulation and analysis code is available upon request.

## Results

### Spatial group size and number

**Fig 2:**
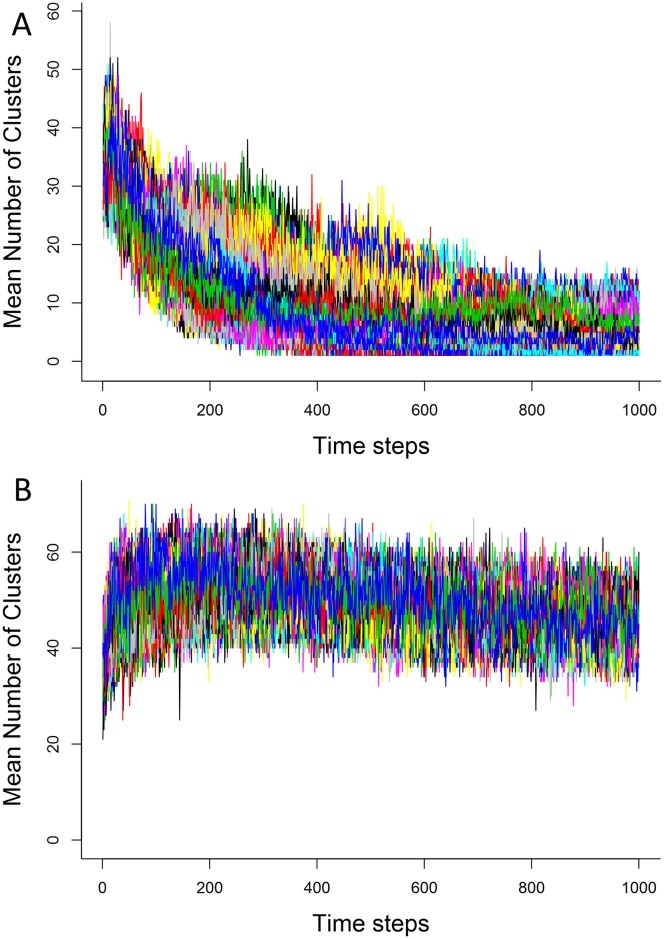
Mean number of clusters without (A) and with (B) memory. Each colored line represents the average number of clusters in a single replicate, total of 100 replicates. World size = 79 units, 1000 agents, memory in A = 20 timesteps, memory modifier = 5%. This shows the effect of IR on aggregation: more groups are formed when individuals recognize each other than with only random interactions.

**Fig 3:**
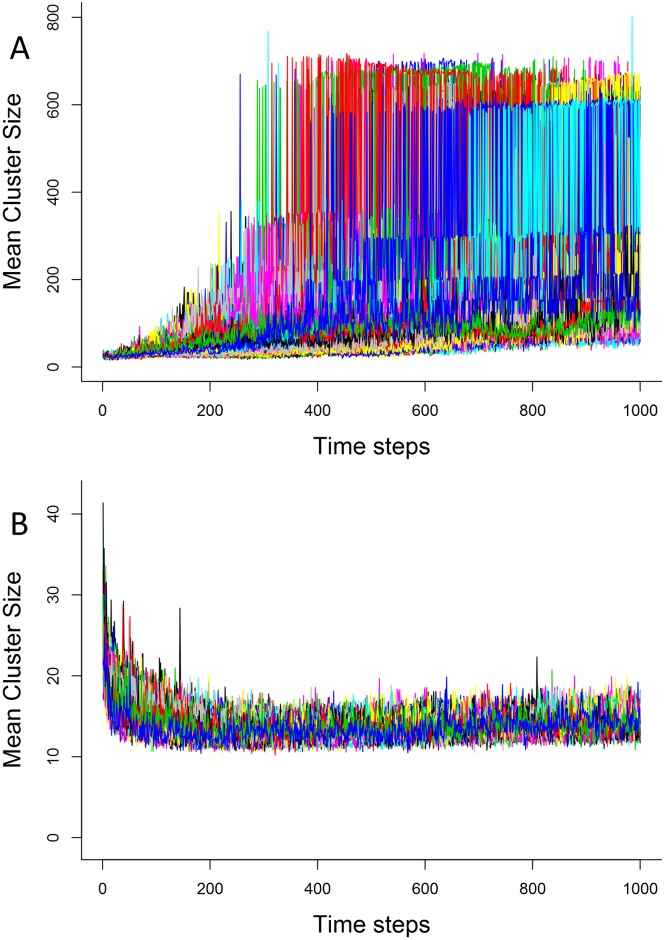
Mean size of clusters without (A) and with (B) memory. Each colored line represents the average number of clusters in a single replicate, total of 100 replicates. World size = 79 units, 1000 agents, memory in A = 20 timesteps, memory modifier = 5%. Average group size differs dramatically when IR is introduced, remaining relatively constant and low, while it increases and varies dramatically when there is no IR (note the different scales on the y axes). This shows the effect of IR on aggregation: smaller groups are formed when individuals recognize each other than with only random interactions.

After a short transient period, the number of groups in simulations without IR decreases dramatically (Fig 2, panel A), from about 30 in the beginning to less than 10 at the end, while it increases in simulations with IR (Fig 2, panel B), from about 30 to around 50 in average. The opposite is seen for average group size (Fig 3), in a much more drastic manner: average group size increases dramatically without IR (Fig 3, panel A), to the point where, in some moments, over half of all agents are in the same cluster (cluster sizes of over 600 in some cases). With IR, group size drops slightly relative to starting conditions(Fig 3, panel B), but in the end are an order of magnitude smaller than the no-IR case. This means that the repeated interactions not only are making the average number of group larger, they are keeping them physically separate, so that we have many small groups at the end of the simulation. Though we detected a difference with the presence of IR, memory intensity did not cause significant differences in group number or size. Group duration was similarly higher in simulations with IR than without.

Population density also has an important effect in spatial grouping. When density is too high, the effects of IR are harder to see, due to limitations of the cluster algorithm: the individuals are too close for DBSCAN to detect distinct groups reliably, and most agents end up being part of a giant cluster (see S1, S2, S3 and S4 Figs). For a matter of consistency, we use the same DBSCAN parameters for all simulations. While using distinct parameters for different densities would possibly detect more groups, these groups would not be comparable between simulations, as they were detected differently. Though this seems an arbitrary limitation, it can be seen as reflecting a real-world situation: when space is an issue, animals will be forced to live closely together, even if they do not interact much.

### Social networks

**Fig 4:**
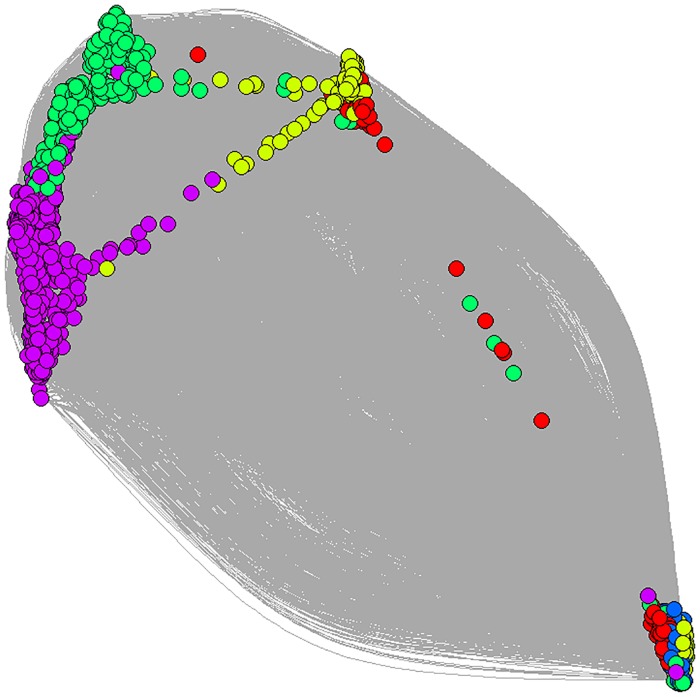
Social network at the end of one simulation. Social network at the end of one simulation. Nodes represent agents; each color represents a different module. Edges are shown in grey. Though edge strength was used to calculate modularity, it is not shown in the graph due to the high number of edges. World size = 79 units, 1000 agents, memory length = 30 time steps, memory modifier = 10%. Nodes in the graph are arranged according to interaction strength: distance between nodes is inversely proportional to the strength of their interactions, i.e., closer nodes have had more affiliative interactions. Distances in the graph are not representative of spatial distances in the simulations.

**Fig 5:**
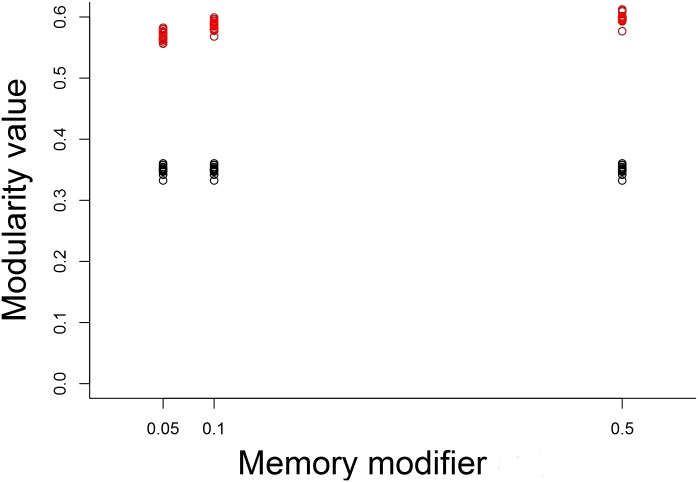
Modularity values without (A) and with (B) IR. Modularity values without (red) and with (black) IR. N = 100 replicates for each. World size = 79 units, 1000 agents, memory in A = 20. Values in red differ significantly from each other and from black ones, *p*<0.01, Kruskal-Wallis test and Dunn’s multiple comparison test. Black values do not differ significantly from each other.

IR also had an effect on network modularity (Figs 4 and 5). Modularity was slightly lower in simulations without IR than in those with IR, meaning that the groups found were more tightly linked. Here we also observed an effect, though slight, of memory intensity: modularity increased slightly when memory intensity was higher.

## Discussion

To approach the question “how do memory and individual recognition affect group structure and stability” we face serious problems: it is difficult to isolate the effects that memory has on social behavior from those caused by other factors, such as resource availability, age and reproductive state. It can also be extremely difficult to determine whether a species exhibits individual recognition or is merely able to determine whether a conspecific falls into a general class (see [26] for a treatment of this problem). Further, if we were to examine the effects of IR, it would be tremendously useful to be able to turn it off and on, to compare the effects of its presence with those of its absence. Though there are methods to experimentally alter the levels of affiliation individuals from a given species exhibit [27], there is currently no way to do this with individual recognition, and doing so could raise ethical questions about the use of transgenic animals. Thus, we use computational modelling to investigate memory and individual recognition, instead of using traditional experiments with live animals.

These simulations indicate that IR does indeed affect the structure of groups. The preferential interactions between individuals result in higher modularity than in simulations without IR, and groups are more spatially discrete. Thus, even though external pressures, which can result in increased densities, can lead to increased grouping, groups created by social behaviors have intrinsically different social structures.

Our results also show that when IR is coupled with preferential interaction, it can result in smaller group sizes than when there is no recognition. [28] discuss that social recognition (and, by extension IR) should only be advantageous in small groups, as the formation and retention of individual memories quickly becomes too costly, and therefore IR should be under negative selection in large groups, being superseded in fitness gains by quality signaling. Indeed, in some of the largest known groups, such as honeybee hives and fish swarms, there is no evidence of individual recognition. We show here that the presence of IR can be a driver for small group sizes without invoking any costs whatsoever, as our model includes no form of fitness payoff or energy expenditure. This is due to the fact that in our model IR results in a positive feedback loop of agonistic and affiliative behaviors: agents who have had an agonistic encounter will tend to remain separate, helping to define spatial groups.

Studying IR can help answer several questions about sociality and animal interactions, for instance, how animals decide whether or not to aggregate or share resources when confronted with resource depletion. Recognition of familiar individuals could increase the probability of repeated interactions, giving rise to forward-feedback phenomena such as reciprocal altruism, and to colony formation. Another more practical application of IR and social memory studies would be in conservation of endangered animals. Fauna translocations are often used to mitigate ecological impacts, but if this is done without consideration for the social systems of the affected species, it can do more harm than good. Introducing foreign individuals to established groups can cause the introduced individuals to be rejected or killed, reducing the success of the translocation, as would separating individuals from their established groups. Knowing whether a species aggregates because of space or resource constraints, or due to social interactions would help plan the translocation or reintroduction of individuals to minimize harm.

This work does not take into account factors like resources, reproduction or fitness, thus providing a null model of the effects of individual recognition in a fixed population. We believe that this type of model can be a powerful resource to compare with other models and experiments. Simulations allow us to investigate aspects of our systems that would otherwise be hard to approach experimentally, and the ability to isolate and manipulate the mechanisms and processes of interest is especially useful. Null models such as this can serve to generate null hypotheses which could be used to design traditional experiments and field investigations, as [29] have done. The use of agents and movement to create an interaction network is a novel and promising approach in animal behavior studies, and allows for a wide range of scenarios to be investigated.

Here, our simulations were identical, except for the presence or absence of IR. If we were to do a traditional experiment to try and answer our questions, we would either have to resort to using closely related, but ultimately different, species, or to come up with a way to “turn off” individual recognition in our test subjects. Either approach would bring about problems and confounding variables. Turning off IR would mean blocking memory formation, retention, and/or recollection, either chemically or via genetic modification, but this could affect other important social behaviors or other types of memory. Infusion of oxytocin, for instance, has been shown to increase duration of social memories [30], but oxytocin and its non-mammalian analogues also affect other social and non-social behaviors [31, 32], which would affect group structure in unknown and possibly uncontrollable ways. Using related species is ideal for studying how IR evolved from a non-IR situation, as [33] have done with Polistine wasps, but even closely related species can have highly different social patterns, as chimpanzees and bonobos [34], which would complicate analysis. Thus, this comparative approach could be unsuitable for many species. Using the example of faunal translocation above, it would be relatively simple to modify the simulations to have the agents moving on a GIS map, and after the groups are established, to add new individuals into the simulation to see whether the groups remain stable or if the introduced individuals are rejected. Fine-tuning of the simulation parameters to match the species of interest would not be difficult, since it would be mostly adjusting the action probabilities to match a more social, solitary or aggressive species, and creating pre-established groups, by creating agents with pre-built memory histories. This simulation framework could also be modified to investigate the evolution of social traits. Here IR is a binary phenomenon, it either exists or not, but a more complex approach could be made by breaking IR into its basic components, (phenotypical variation of identity cues, perception of these cues, and the action taken based on this perception, see [35]), and making these components variable and inheritable in the simulation. Another approach would be to investigate if class-level recognition leads to different group structures than individual recognition, or if IR and class-leve recognition are functionally the same after a certain group size

## Supporting Information

S1 Text ODD Protocol. Contains a detailed description of the model and analysis algorithms used.
(PDF)

S1 Fig Effects of high density. Panels A and B show that at high densities, all individuals are forced into a single cluster most of the time. Each colored line represents one replicate, total of 10 replicates. World size = 45 units, 1000 agents, memory in A = 20 timesteps, memory modifier = 5%.
(TIF)

S2 Fig Effects of low density. Panels A and B show that at low densities, no clusters form, as individuals are too spread out. Note that the minimum size for a cluster to be detected with DBSCAN is 4 individuals. Each colored line represents one replicate, total of 10 replicates. World size = 250 units, 1000 agents, memory in A = 20 timesteps, memory modifier = 5%.
(TIF)

S3 Fig Effects of shorter memories. Panels A and B show Mean cluster size and mean number of clusters. Each colored line represents one replicate, total of 10 replicates. World size = 79 units, 1000 agents, memory in A = 2 timesteps, memory modifier = 5%. The same pattern is seen in Figs 2 and 3 of the manuscript, showing that the mere presence of IR is sufficient to induce group formation.
(TIF)

S4 Fig Distribution of group sizes without (A) and with (B) memory. Total of 100 replicates. World size = 79 units, 1000 agents, memory in A = 20 timesteps, memory modifier = 5%. Group sizes were polled from 100 replicates, taken at the end of the simulations This shows the effect of IR on aggregation: more, smaller groups are formed when individuals recognize each other (A) than with only random interactions (B), where more varied group sizes are seen. The same pattern is seen in Figs 2 and 3 of the main paper, showing that the mere presence of IR is sufficient to induce group formation.
(TIF)

## References

[1] Alexander RD. The evolution of social behavior. Annu Rev Ecol Evol Syst. 1974;5(4):325–383. 10.1162/106454603322694861

[2] Sherman PW, Lacey EA, Reeve HK, Keller L. The eusociality continuum. Behav Ecol. 1995;6(1):102–108. 10.1093/beheco/6.1.102

[3] Hamilton WD. Geometry for the selfish herd. J Theor Biol. 1971;31(2):295–311. 10.1016/0022-5193(71)90189-5 5104951

[4] Clark CW, Mangel M. The evolutionary advantage of group foraging. Theor Popul Biol. 1986;30(1):45–74. 10.1016/0040-5809(86)90024-9

[5] Ebensperger LA, Cofré H. On the evolution of group-living in the New World cursorial hystricognath rodents. Behav Ecol. 2001;12(2):227–236. 10.1093/beheco/12.2.227

[6] Hildenbrandt H, Carere C, Hemelrijk CK. Self-organized aerial displays of thousands of starlings: a model. Behav Ecol. 2010;21(6):1349–1359. 10.1093/beheco/arq149

[7] Lacey EA, Wieczorek JR. Ecology of sociality in rodents: a ctenomyid perspective. J Mammal. 2003;84(4):1198–1211. 10.1644/BLe-014

[8] Kerth G, Perony N, Schweitzer F. Bats are able to maintain long-term social relationships despite the high fission-fusion dynamics of their groups. Proc R Soc B Biol Sci. 2011;278(1719):2761–2767. 10.1098/rspb.2010.2718 21307051 PMC3145188

[9] Johnson DDP, Kays R, Blackwell PG, Macdonald DW. Does the resource dispersion hypothesis explain group living? Trends Ecol Evol. 2002;17(12):563–570. 10.1016/S0169-5347(02)02619-8

[10] Johnstone RA. Models of reproductive skew: A review and synthesis. Ethology. 2000;106(1):5–26. 10.1046/j.1439-0310.2000.00529.x

[11] Nowak MA, Bonhoeffer S, May RM. Spatial games and the maintenance of cooperation. PNAS Proc Natl Acad United States Am. 1994;91(11):4877–81. 10.1073/pnas.91.11.4877 PMC43892 8197150

[12] Tibbetts EA, Dale J. Individual recognition: it is good to be different. Trends Ecol Evol. 2007;22(10):529–37. 10.1016/j.tree.2007.09.001 17904686

[13] Breed M. Recognition Pheromones of the Honey Bee. Bioscience. 1998;48(6):463–470.

[14] Grimm V, Railsback S. Individual-based Modeling and Ecology. Princeton: Princeton University Press; 2005 10.1515/9781400850624

[15] Martins AB, Aguiar MAMD, Bar-yam Y. Evolution and stability of ring species. PNAS: Proceedings of the National Academy of Sciences of the United States of America. 2013;110(13):5080–5084. doi: 10.1073/pnas.1217034110/-/DCSupplemental . 10.1073/pnas.1217034110/-/DCSupplemental www.pnas.org/cgi/doi/10.1073/pnas.1217034110. www.pnas.org/cgi/doi/10.1073/pnas.1217034110 PMC3612659 23479635

[16] Grimm V, Revilla E, Berger U, Jeltsch F, Mooij WM, Railsback SF, et al Pattern-oriented modeling of agent-based complex systems: lessons from ecology. Science. 2005;310(5750):987–91. 10.1126/science.1116681 16284171

[17] Hemelrijk CK. Towards the integration of social dominance and spatial structure. Anim Behav. 2000;59(5):1035–1048. 10.1006/anbe.2000.1400 10860531

[18] Grimm V, Berger U, DeAngelis DL, Polhill JG, Giske J, Railsback SF. The ODD protocol: A review and first update. Ecol Modell. 2010;221(23):2760–2768. 10.1016/j.ecolmodel.2010.08.019

[19] Ester M, Kriegel HP, Sander J, Xu X. A density-based algorithm for discovering clusters in large spatial databases with noise. In: Proc. 2nd Int. Conf. Knowl. Discov. Data Min.; 1996. p. 226–231. Available from: http://www.aaai.org/Papers/KDD/1996/KDD96-037.pdf.

[20] Spiliopoulou M, Ntoutsi I, Theodoridis Y, Schult R. Monic: modeling and monitoring cluster transitions. In: Proc. 12th ACM SIGKDD Int. Conf. Knowl. Discov. data Min.; 2006. p. 706–711. Available from: http://dl.acm.org/citation.cfm?id=1150491.

[21] Newman MEJ. Structure and function of complex networks. SIAM Rev. 2003;45(2):167–256. 10.1137/S003614450342480

[22] Newman MEJ. Modularity and community structure in networks. PNAS Proc Natl Acad United States Am. 2006;103(23):8577–82. 10.1073/pnas.0601602103 PMC1482622 16723398

[23] Blondel VD, Guillaume Jl, Lambiotte R, Lefebvre E. Fast unfolding of communities in large networks. J Stat Mech Theory Exp. 2008;10008(10):6 10.1088/1742-5468/2008/10/P10008

[24] R Core Team. R: A Language and Environment for Statistical Computing; 2015 Available from: http://www.r-project.org/.

[25] Csárdi G, Nepusz T. The igraph software package for complex network research. InterJournal Complex Syst. 2006;1695:1695.

[26] Gherardi F, Aquiloni L, Tricarico E. Revisiting social recognition systems in invertebrates. Anim Cogn. 2012;15(5):745–62. 10.1007/s10071-012-0513-y 22639070

[27] Young LJ, Nilsen R, Waymire KG, MacGregor GR, Insel TR. Increased affiliative response to vasopressin in mice expressing the V1a receptor from a monogamous vole. Nature. 1999;400:766–8. 10.1038/23475 10466725

[28] Sheehan MJ, Bergman TJ. Is there an evolutionary trade-off between quality signaling and social recognition? Behav Ecol. 2016;27(1):2–13. 10.1093/beheco/arv109

[29] King AJ, Sueur C, Huchard E, Cowlishaw G. A rule-of-thumb based on social affiliation explains collective movements in desert baboons. Anim Behav. 2011;82(6):1337–1345. 10.1016/j.anbehav.2011.09.017

[30] Dluzen DE, Muraoka S, Engelmann M, Ebner K, Landgraf R. Oxytocin induces preservation of social recognition in male rats by activating alpha-adrenoceptors of the olfactory bulb. Eur J Neurosci. 2000;12(2):760–766. 10.1046/j.1460-9568.2000.00952.x 10712656

[31] Gruber CW. Physiology of invertebrate oxytocin and vasopressin neuropeptides. Exp Physiol. 2014;99(1):55–61. 10.1113/expphysiol.2013.072561 23955310 PMC3883647

[32] Donaldson ZR, Young LJ. Oxytocin, vasopressin, and the neurogenetics of sociality. Science. 2008;322:900–904. 10.1126/science.1158668 18988842

[33] Sheehan MJ, Straub MA, Tibbetts EA. How Does Individual Recognition Evolve? Comparing Responses to Identity Information in Polistes Species with and Without Individual Recognition. Ethology. 2014;120:169–179. 10.1111/eth.12191

[34] Palagi E. Social play in bonobos (*Pan paniscus*) and chimpanzees (*Pan troglodytes*): Implications for natural social systems and interindividual relationships. Am J Phys Anthropol. 2006;129(3):418–426. 10.1002/ajpa.20289 16323189

[35] Mateo JM. Recognition systems and biological organization: The perception component of social recognition. Ann Zool Fennici. 2004;41(6):729–745.

