# Supplementary material for: Do I Know You? How Individual Recognition Affects Group Formation and Structure
Source: PLoS One. 2017 Jan 26;12(1):e0170737. doi: 10.1371/journal.pone.0170737 (PMC5268392; doi:10.1371/journal.pone.0170737)
Supplement: S1 Text — Contains a detailed description of the model and analysis algorithms used. (PDF) [file pone.0170737.s001.pdf]

ODD Protocol
SocS – Social Simulator

This is the OOD protocol describing our model, as proposed by [1]. It is intended as a verbal description of the model to allow understanding and re-implementation of the code if necessary.The full source code for our model is available upon request.

If we are to study sociality and group behavior, it is important to clarify exactly what we mean by "social behavior", "group", "memory" and other terms, so that we can properly analyze and model them, especially because this field of study is fraught with multiple, conflicting definitions (see [2], for an analysis of this problem).

Social behaviors are defined here rather loosely as "any interaction between two conspecifics". Though this definition is certainly not a consensus, it is useful and sufficient for our purposes, as it avoids biases towards "socio-positive" behaviors such as allogrooming and mating, and includes aggressive behaviors, which are usually not seen as "social", but affect a group's structure and stability. We divide social behaviors in two categories, based on their long-term effects on the group: *affiliative behaviors* are "behaviors which tend to increase grouping" and *agonistic behaviors* are "behaviors which tend decrease grouping". Thus, mating and food sharing are affiliative behaviors, in that the proximity they cause is long lasting. A fight is considered an agonistic behavior because, while it certainly brings individuals together for the duration of the event, it is safe to say that at least one of the animals will leave the area afterwards, especially in territorial disputes (unless one of the animals perishes). *Interactions* and social behaviors are used interchangeably here. These definitions are based purely on movement, to reflect our definition of group. All behaviors that do not involve interaction with other conspecifics directly, that is, which do not affect the group, are lumped together as *neutral behaviors*. Neutral behaviors can bring individuals closer or apart, but this proximity is not derived from interaction, and therefore not social under our definitions. As our definition of group is not based on social interactions, neutral behaviors can give rise to groups, but it is expected that these groups will be short-lived, as the non-interacting individuals go about their businesses. These definitions may appear to be oversimplifications of complex phenomena, and it would certainly be more realistic to add gradations to the intensity of each type of behavior, but doing so would add unnecessarily complexity to the model. A simple

affiliative/agonistic/neutral classification of behaviors allows us to compare situations with and without memory without worrying about the effects of affiliation or aggression levels, or the external causes of these behaviors.

Since we are interested on the effects of repeated interactions, definitions of memory and individual recognition as they pertain to social behaviors are also required. Here, *memory* is defined as "an individual's record of its previous interactions (*social behaviors*) with other conspecifics" and *individual recognition* (IR) is "the ability to associate a particular memory with a particular conspecific". We will use the terms *memory* and *social memory* interchangeably. Though other types of memory are important for the individual's survival, we will not concern ourselves with them here. The mechanisms by which individuals recognize each other and by which memories are formed are not relevant for this paper. We will use these basic definitions to create a model of social memory to investigate its effects on group formation and structure.Simulating perfect memory, that is, remembering all previous encounters with all other agents would be computationally prohibitive due to the size and length of the simulations.

1. Purpose

   What is the purpose of the model?

   This model intends to explore the effects of memory and individual recognition on group formation and structure. In other words, we review how the effect of past interactions affect the structure and stability of groups in a population of agents.

2. Entities, state variables, and scales

   What kinds of entities are in the model? By what state variables, or attributes, are these entities characterized? What are the temporal and spatial resolutions and extents of the model?

   The only two entities present are the world and the agents. The world exists as a proxy for space, and its only actions are to define the order of interactions, and to keep a register of the agents' spatial coordinates. In all simulations the world is represented by the surface of a torus, that is, a 2d space with continuous boundaries, homogeneous, and without corners. The size of the world varies between to simulate different agent densities.

   Each agent is a separate individual, representing a freely moving animal with independent behaviors. The agents are points in space, and have only 6 important traits: *position, perception radius, step size, action probabilities*, and *memory.*

   (a) *Position* is the agents X and Y coordinates

(b) Perception radius is how far in space the agents can perceive one another.

(c) *Maximum step size* is the maximum distance an agent can move in a single action. A single step is a random length between 0 and *maximum step size*.

(d) *Action Probability* describe how likely an agent is to engage in one of three behaviors when in a social situation: affiliative, agonistic and neutral.

(e) *Memory* is a record of the previous interactions each agent has had with other agents, and described by *memory length*, the maximum number of interaction an agent can remember, and *memory intensity*, how strongly each remembered interaction affects the agents' action probabilities (see below). All agents are initially identical, differing only in their initial coordinates, and all have empty initial memories

(f) *Space* is measured in arbitrary units, as time is measured in rounds. All agents act only once per round. Each action is a step and a possible interaction with a single other agent. See below for details of interactions

3. Process overview and scheduling

Who (i.e., what entity) does what, and in what order? When are state variables updated? How is time modeled, as discrete steps or as a continuum over which both continuous processes and discrete events can occur?

On each round, each agent is called to act and possibly interact with the surrounding agents. The calling order is randomized at the beginning of each round, and all agents are called once per round. Each agent has access to the current state of the world around it, with the updated positions after the previous agent has acted. This asynchronous updating simulates a continuous time process [3]. Each round occurs as follows:

(a) Round starts, World randomizes order of agents

(b) Each agent (called *actor* when on its turn to act) performs the following procedures once. All agents act, one after the other, in the order determined in step 1

i. Actor searches perception radius for other agents

A. If there are any, choose the one with whom actor has the highest affiliative memory and proceed to the *encounter* sub-process, and both agents' memories are updated accordingly

B. If no other agents in perception radius, actor takes a step of random length (from 0 up to *step size*) in a random direction

ii. Actor position is updated on the world

(c) Round ends, statistics are collected, proceed to next round.

As actor positions are updated constantly, each agent has access to the current state of the world. This approximates a continuous time, even though round are discrete and non-overlapping.

If actor finds at least one other agent in its *perception radius*, it chooses the one with whom it has the highest count of affiliative events in its memory. If more than one has the same count, one is picked at random. This is intended to mimick the phenomenon where affiliative memory induces preferential interactions, that is, given a choice, all things being equal, animals will prefer to interact with familiar animals. Test simulations have shown that when the actor chooses a partner at random, the effects of recognition are diminished. The *encounter* sub-process is described in Submodels, below.

4. Design Concepts

(a) Basic Principles

The most basic principle in this model is that memory has an effect on an individual's decision on how to behave relative to familiar individuals. (See main paper for reasoning behind these behaviors)

When confronted with another individual, an actor has three options: it can ignore the other, a behavior we call *neutral*; it can engage in an *agonistic behavior*, e.g. an aggressive display or a fight, or it can engage in *affiliative behaviors*, such as grooming or sharing resources. We assume that each of these social behaviors has a different consequence on group formation and maintenance: *Neutral* behaviors do nothing at all for the group. The actor proceeds as if there had not been an encounter. *Affiliative* behaviors increase group cohesion, that is, the actor steps closer to the other agent. *Agonistic* behaviors are the opposite, that is, the actor steps away from the other agent

(b) Emergence The group or groups emerge from the interactions of individuals agents. A group is determined spatially as a cluster of individuals using the definitions of clusters from the DBSCAN algorithm: groups of points, which are density-connected, that is, are within a

certain distance from at least one other member of the cluster. See the DBScan algorithm section for details.

Spatial clusters are calculated at the end of every round. After the simulations ends, the clusters from each round are compared using the MONIC algorithm

(c) Adaptation

Agents react to the presence of other agents based on the memory of previous interactions. They can ignore, approach, or avoid other agents, depending on their memory, with a cumulative effect

(d) Objectives

Agents do not have an explicit objective. They move randomly until they meet another agent. Even when meeting another agent, they act based on an action probability, not an explicit intention

(e) Learning

Individuals learn what type of interaction they had, and the identity of the agents with whom they interacted. Memory also has a definite length, after which older interactions no longer affect the outcome of the current interaction

(f) Prediction

Agents are not capable of prediction

(g) Sensing

Agents can sense the presence of another if they are within their perception radius. They can perceive the others' identities and remember previous interactions. They always choose to interact with the individual with whom they had more affiliative interactions.

(h) Interaction

The agents can approach, avoid or ignore each other. If they have an affiliative interaction, the actor approaches the target agent. In case of an agonistic interaction, the agent moves away from the target agent, and if the action is neutral, the agents moves randomly. The type of interaction is chosen based on the actor's action probabilities, modified by its memory

Each actor can only interact with one other agent on each round

(i) Stochasticity

The interactions are based on action probabilities. Each agent has an initial probability of 1/3 for each action, affiliative, agonistic or neutral. This means that if there is no effect from memory (if the agents have never met) the type of interaction is random, with equal probabilities. These probabilities are modified according to the memory submodel below, but are always between zero and one (not inclusive), that is, even if the agents have always acted afilliatively, there is a small chance they can act agonistically or neutrally.

(j) Collectives

There are no explicit collectives. Clusters are calculated at every step, but have no bearing on an individual's action. The individual has no concept clusters or grouping.

(k) Observation

At the end of each round, spatial clusters are computed via DBSCAN, and a register is kept of who interacted with whom and how (what type of action was performed). At the end of the simulation, the MONIC algorithm is run to calculate lifetime of the clusters. The sum of the actions between two agents is also calculated (affilitives are counted as +1, agonistics as -1 and neutrals as 0), and this is used to investigate the social networks formed between the agents. The number of clusters and average cluster size is also registered for each round

5. Initialization

At time t=0, the agents are distributed randomly throughout the world. All agents are identical and all memories are empty. There are 1000 agents, but the world size varies to simulate different densities. Interaction radius is 3 units, step size is 2 units. Epsilon and MinPts (see DBSCAN below) are 3 and 4, respectively. These values were taken from [4], which describes these values as being adequate for two-dimensional spaces. Memory length and effect size are also set at initialization, but vary between simulations

6. Input data

There is no external input data other than the parameters described above

7. Submodels

(a) Action

Actions are modeled as movement. Since we define a group as an aggregation of individual which persists in time, affiliative actions are defined as those that aggregate individuals, and

agonistic action are define as those which tend to segregate individual. Neutral actions are defined as not having a direct impact on grouping and therefore are represented by random motion. If there no other agents on an actor's perception radius, then it moves in random direction, to a distance of up to its maximum step size.

After an affiliative encounter, the actor takes a step in the direction of the target agent. Because step size is random (between 0 and *stepsize*), and can be slightly greater than the distance between the agents, they do not converge on a single spot a sequence of affiliative interactions. After an agonistic encounter, the actor takes a step in the opposite direction

(b) Memory

The agent's memory is composed of a list of interactions with a given *memory length*. This length is fixed at the start of the simulation. Each agent has a different list for each agent whit whom it has interacted. Interactions are added to this list, and once the maximum length is reached, the oldest interaction is removed to make space for the newest one. The three types of action are added to the same list. This implies that an individual's memory towards another can change with a sequence of interactions.

Memories also have an *intensity*, that is, the amount that is added to the agent's initial action probability to determine the outcome of the encounter. This intensity is assumed constant and equal for all interaction types. The three action probabilities that must always add up to one, and can never be zero (the minimum value for each is 0.05%, and the maximum 99,9%).

(c) Encounter

The encounters are determined in the following way: the actor scans its memory for previous interactions with each other agent in its interaction radius, and chooses the one with whom it has the highest affiliative modifier. Action modifiers are calculated according to the following formulae:

$Affiliative\ modifier(af) = total\ Affiliative\ actions * intensity\ modifier$

$Agonistic\ modifier(ag) = total\ Agonistic\ actions * intensity\ modifier$

$Neutral\ modifier(an) = total\ Neutral\ actions * intensity\ modifier$

Once the target agent is chosen, the action probabilities are calculated as follows:

$$Affiliative = \frac{1}{3} + af - \frac{ag}{2} - \frac{an}{2} \tag{1}$$

$$Affiliative = \frac{1}{3} + ag - \frac{af}{2} - \frac{an}{2} \quad (2)$$

$$Affiliative = \frac{1}{3} + an - \frac{af}{2} - \frac{ag}{2} \quad (3)$$

To calculate the affiliative probability, we add the affiliative modifier to the base action probability and subtract half the agonistic modifier and half the neutral modifier. This is because these probabilities are complimentary: increasing one must forcefully decrease the other two in equal measure, and they must always add up to one. The same is done for the other probabilities: the final value is the sum of its modifier, minus half of each of the modifiers of the other actions. These probabilities are never allowed to exceed 99,9% or to go below 0.05%. This is to reflect the fact that even though two animals can have extremely high affiliation levels, there is still the possibility of agonistic or neutral events, and the same holds true for levels of agonism and neutrality.

The type of action to be performed is then drawn from these probabilities by using an uniform distribution random number generator. Once the type of action is determined, the actor acts as follows: if the action is affiliative, the actor and the target move toward each other. If it is agonistic, both move away from each other. If the action is neutral, the actor moves in a random direction. Both the actor and the target then register the interaction in their memories.

(d) The positions of all agents are fed into the analyses algorithms (see below)

1 Analysis Algorithms

1.1 DBSCAN

[4] Though not part of the simulation *per se*, *DBSCAN* and *MONIC* are an important part of the model results' analyses and are therefore included here. DBSCAN is a clustering algorithm that happens to be a close match to the group definition we use in this paper: clusters are defined by a minimum density and can be any shape or size beyond a certain minimum, and points can also be noise, that is not belong to any cluster. It is dependent on two parameters: *Epsilon*, the neighborhood radius of a point; and *MinPts*, the minimum number of points that have to be inside a given point's *Epsilon* for the set (center point plus neighbors) to be considered a cluster. In this paper, each agent is assumed to be a point for clustering purposes.

This procedure returns a set of clusters with its member points, and the points that are not members of any cluster (noise). The results of this clustering for each point in time are the processed by the MONIC algorithm to investigate the duration of the groups. The algorithm is as follows:

1. For each point:
   (a) Check neighborhood in range *Epsilon*.
   (b) If there are less neighbors than *MinPts*, point is considered noise.
   (c) If there are more neighbors than *MinPts*, a cluster is found Each point found in this cluster is marked as part of the cluster, and its *Epsilon* neighborhood is searched, and any neighbors found are also marked as part of the cluster. This is repeated for all points found inside all *Epsilon* neighborhoods, until no more new points are found in the *Epsilon* neighborhood of any member of the cluster.
   (d) Proceed to next unmarked point.

1.2 MONIC

[5]

MONIC compares sequential clusterings and finds which clusters have survived in consecutive timesteps, by comparing which sequential groups are composed of at least 50% of the same individuals. Doing this this for all rounds allows us to calculate the average lifetime of groups

References

References

1. Grimm V, Berger U, DeAngelis DL, Polhill JG, Giske J, Railsback SF. The ODD protocol: A review and first update. Ecol Modell. 2010;221(23):2760–2768. doi:10.1016/j.ecolmodel.2010.08.019.

2. Costa JT, Fitzgerald TD. Social terminology revisited: Where are we ten years later? Annales Zoologici Fennici. 2005;42:559–564.

3. Caron-Lormier G, Humphry RW, Bohan DA, Hawes C, Thorbek P. Asynchronous and synchronous updating in individual-based models. Ecological Modelling. 2008;212(3-4):522–527. doi:10.1016/j.ecolmodel.2007.10.049.

4. Ester M, Kriegel HP, Sander J, Xu X. A density-based algorithm for discovering clusters in large spatial databases with noise. In: Proc. 2nd Int. Conf. Knowl. Discov. Data Min.; 1996. p. 226–231. Available from: http://www.aaai.org/Papers/KDD/1996/KDD96-037.pdf.

5. Spiliopoulou M, Ntoutsi I, Theodoridis Y, Schult R. Monic: modeling and monitoring cluster transitions. In: Proc. 12th ACM SIGKDD Int. Conf. Knowl. Discov. data Min.; 2006. p. 706–711. Available from: http://dl.acm.org/citation.cfm?id=1150491.
