# Supplementary figures and images for: Do I Know You? How Individual Recognition Affects Group Formation and Structure
Source: PLoS One. 2017 Jan 26;12(1):e0170737. doi: 10.1371/journal.pone.0170737 (PMC5268392; doi:10.1371/journal.pone.0170737)

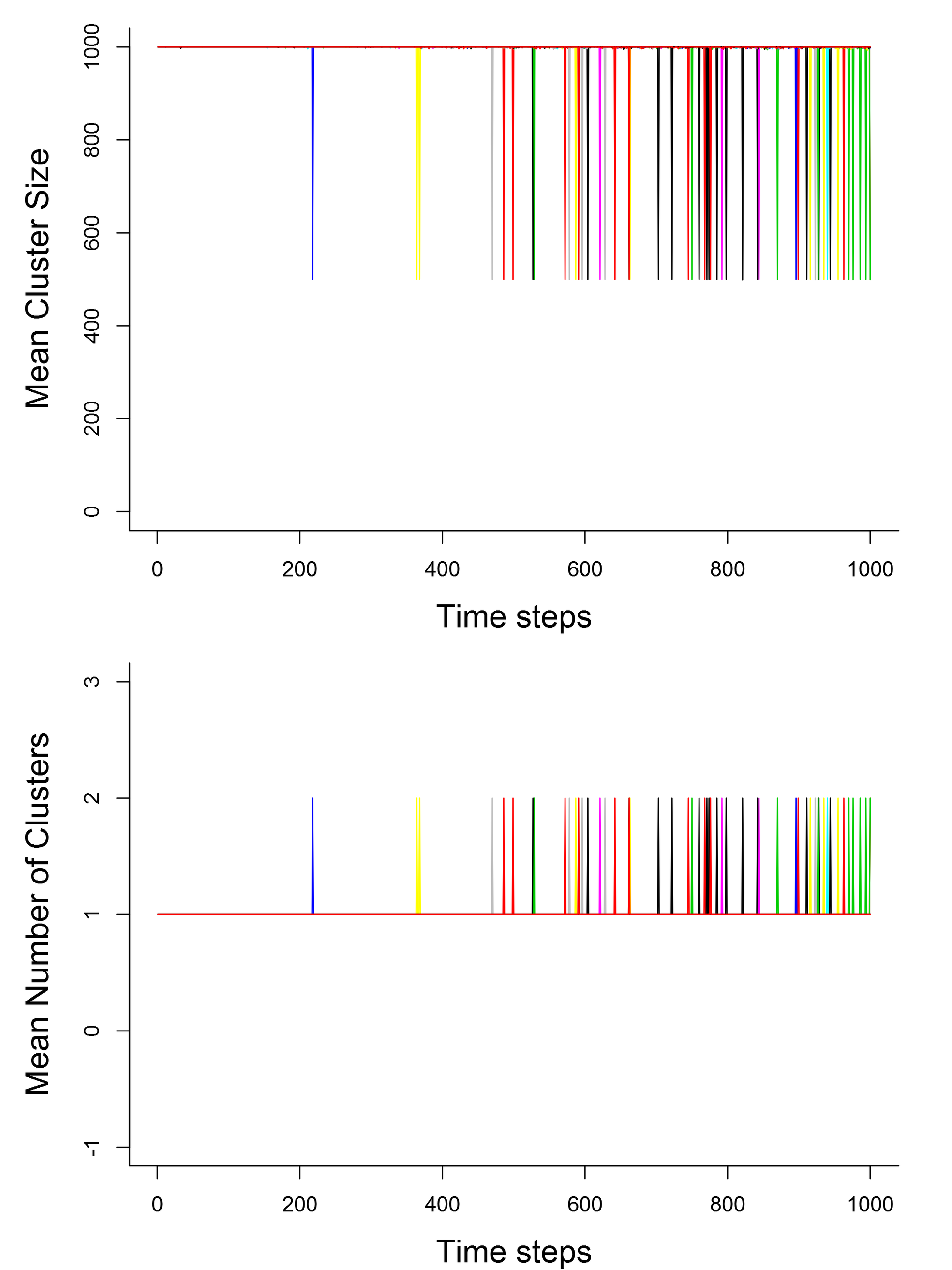

Supplement: S1 Fig — Panels A and B show that at high densities, all individuals are forced into a single cluster most of the time. Each colored line represents one replicate, total of 10 replicates. World size = 45 units, 1000 agents, memory in A = 20 timesteps, memory modifier = 5%. (TIF) [file pone.0170737.s002.tif]

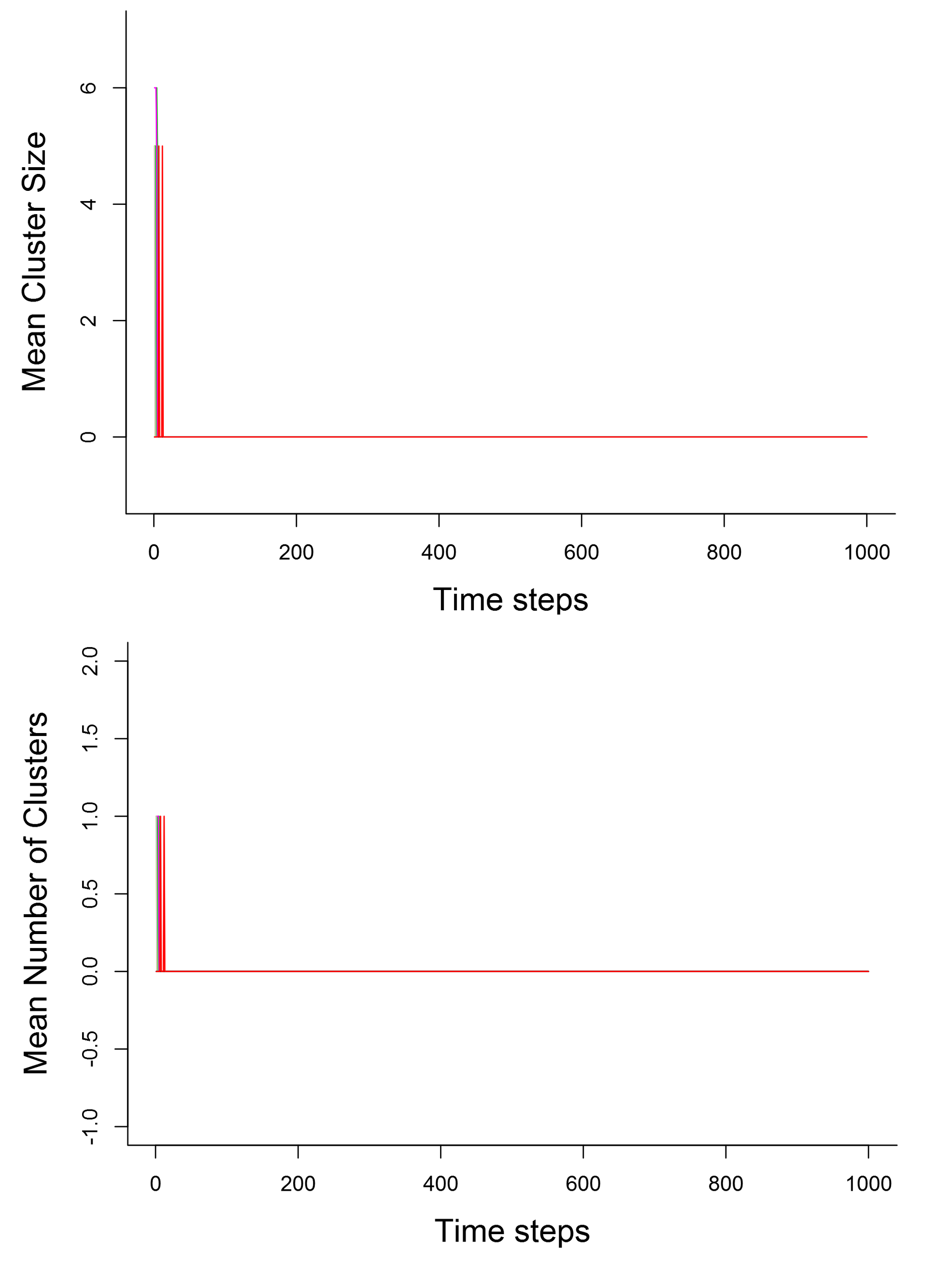

Supplement: S2 Fig — Panels A and B show that at low densities, no clusters form, as individuals are too spread out. Note that the minimum size for a cluster to be detected with DBSCAN is 4 individuals. Each colored line represents one replicate, total of 10 replicates. World size = 250 units, 1000 agents, memory in A = 20 timesteps, memory modifier = 5%. (TIF) [file pone.0170737.s003.tif]

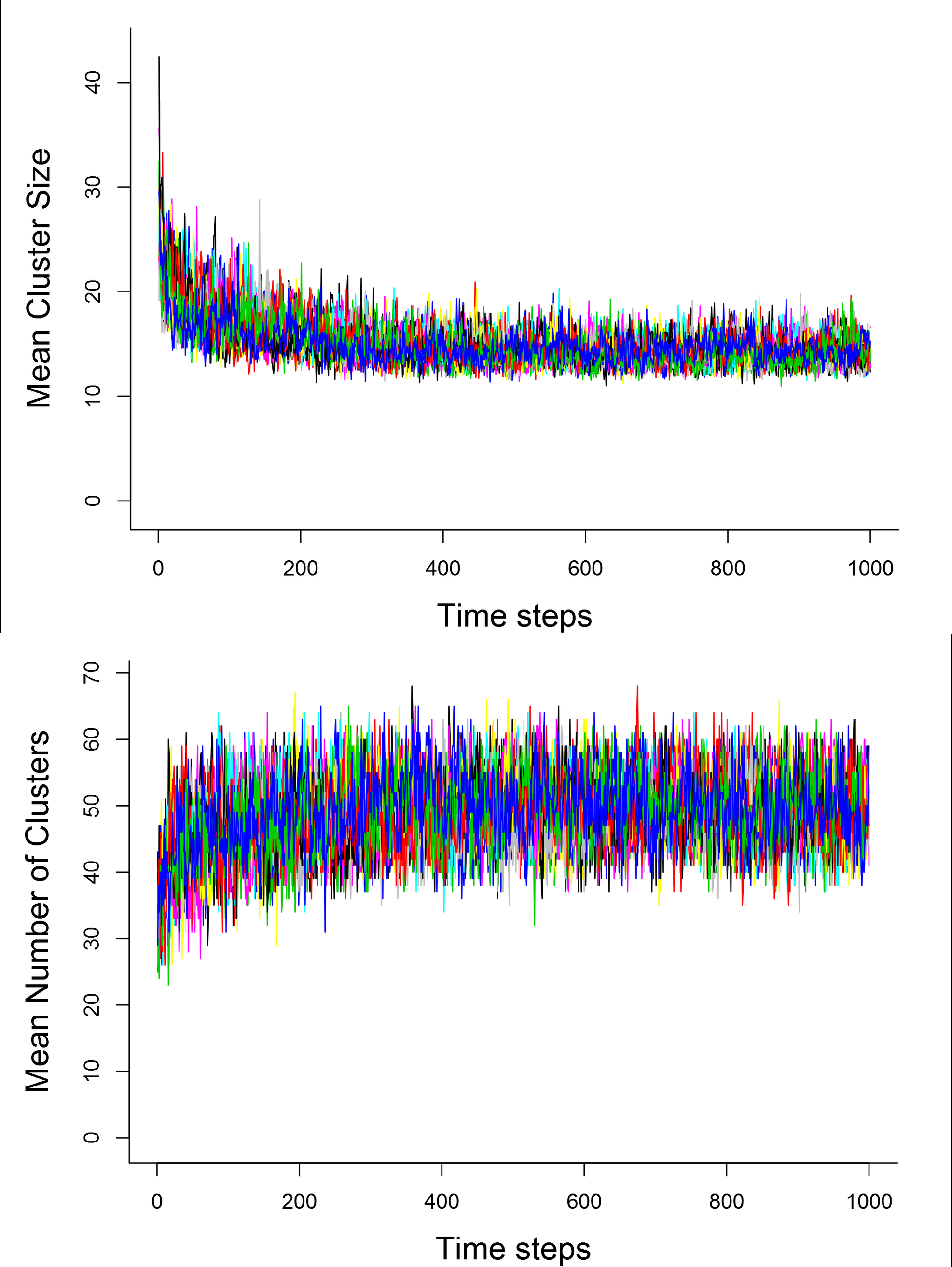

Supplement: S3 Fig — Panels A and B show Mean cluster size and mean number of clusters. Each colored line represents one replicate, total of 10 replicates. World size = 79 units, 1000 agents, memory in A = 2 timesteps, memory modifier = 5%. The same pattern is seen in Figs 2 and 3 of the manuscript, showing that the mere presence of IR is sufficient to induce group formation. (TIF) [file pone.0170737.s004.tif]

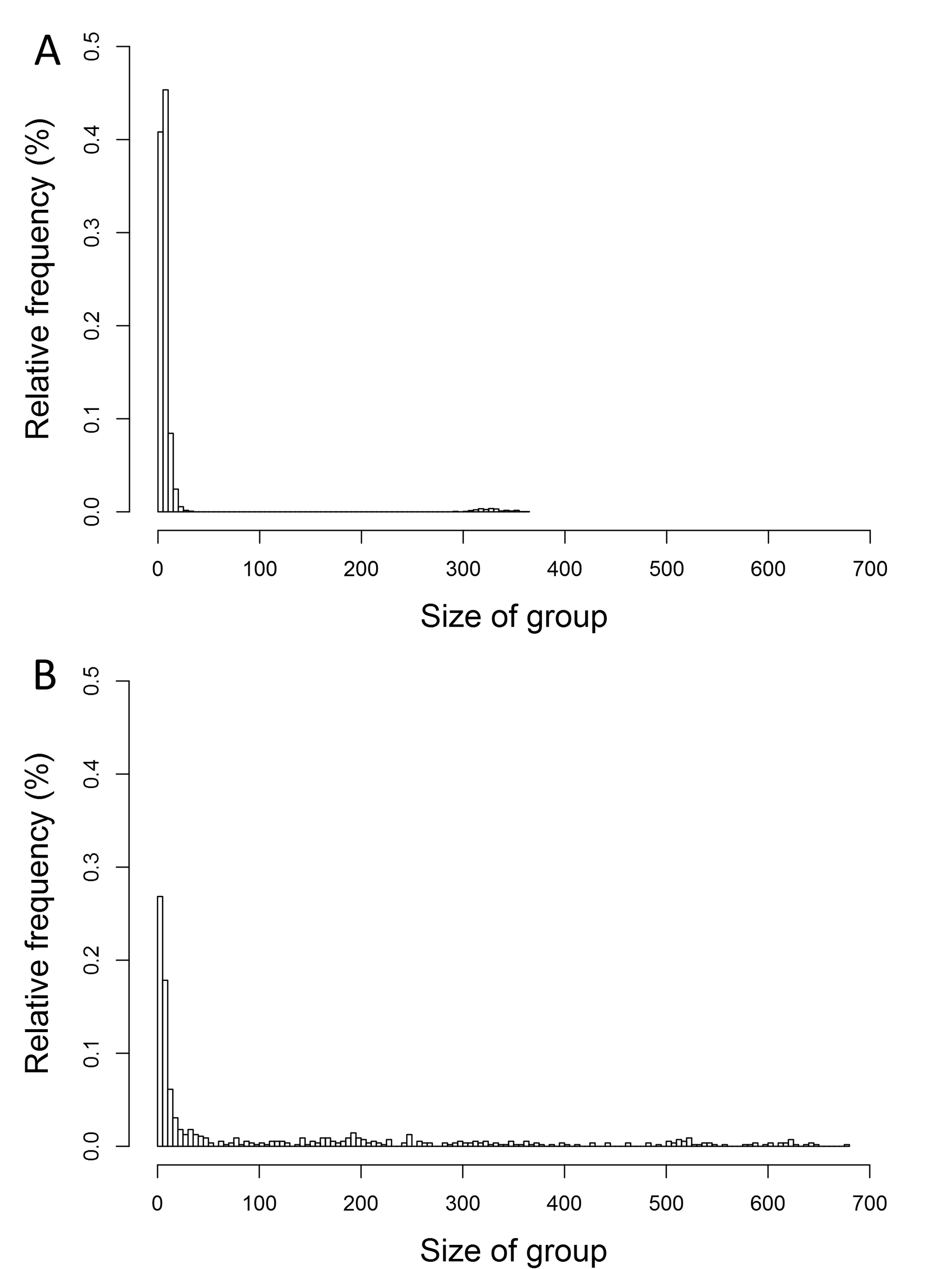

Supplement: S4 Fig — Total of 100 replicates. World size = 79 units, 1000 agents, memory in A = 20 timesteps, memory modifier = 5%. Group sizes were polled from 100 replicates, taken at the end of the simulations This shows the effect of IR on aggregation: more, smaller groups are formed when individuals recognize each other (A) than with only random interactions (B), where more varied group sizes are seen. The same pattern is seen in Figs 2 and 3 of the main paper, showing that the mere presence of IR is sufficient to induce group formation. (TIF) [file pone.0170737.s005.tif]
